# The Whitening, Moisturizing, Anti-aging Activities, and Skincare Evaluation of Selenium-Enriched Mung Bean Fermentation Broth

**DOI:** 10.3389/fnut.2022.837168

**Published:** 2022-03-18

**Authors:** Kang Wei, Congyin Guo, Jiangxiong Zhu, Yang Wei, Meirong Wu, Xiaodong Huang, Mu Zhang, Jide Li, Xueyun Wang, Yuanfeng Wang, Xinlin Wei

**Affiliations:** ^1^School of Agriculture and Biology, Shanghai Jiao Tong University, Shanghai, China; ^2^College of Life Sciences, Shanghai Normal University, Shanghai, China; ^3^Shanghai Yuemu Cosmetics Co., Ltd., Shanghai, China; ^4^Enshi Selenium Impression Agricultural Technology Co., Ltd., Shadi Township, Enshi, China

**Keywords:** Se-enriched mung bean fermentation broth, chemical composition, whitening, moisturizing, anti-aging, facemask

## Abstract

Selenium-enriched mung bean (Se-MB) is a combination of mung bean (MB) and selenium (Se), which have a variety of potential biological activities. However, little is known about the skincare activity of Se-MB. The chemical composition of Se-MB fermentation broth (Se-MBFB) was analyzed to investigate the whitening, moisturizing, and anti-aging activities of Se-MBFB. The tyrosinase inhibition, anti-melanogenic in melanocytes (B16F10 cells), and moisturizing effect in human dermal fibroblasts (HDFs) were analyzed. Besides, the free radical scavenging activity of Se-MBFB was assessed *in vitro*. To verify the *in vivo* effects and the potential of practical applications of Se-MBFB, a clinical trial was conducted on the participants: 31 Chinese women aged 25–60 years, with no pigmentation disorder, no illness, no history of hypersensitivity reaction, and no use of skincare product on the face. The participants used an Se-MBFB masque for 15-20 min after cleaning the face. The measurement points were Week 0, 2, and 4 (W0, W2, and W4) after using the masque, and target sites were cheek and canthus. The following parameters were recorded on the target sites at each visit: melanin index, skin color, cuticle moisture content, transepidermal water loss, and crow's feet. The results demonstrated that Se-MBFB was rich in polyphenols, peptides, and γ-aminobutyric acid (GABA), displayed significant free radical scavenging and tyrosinase inhibiting activities, decreased the synthesis of melanin, and upregulated the aquaporin-3 (AQP3) expression. The test of the Se-MBFB mask showed that after 4 weeks of using the Se-MBFB facemask, the faces of the participants became whiter with reduced wrinkles and increased moisture content. Se-MB possessed the excellent whitening, moisturizing, and antioxidant efficacy, which could lay a scientific foundation for utilization and development of skincare products of Se-MB and its related industrial cosmetics products.

## Introduction

The skin is the largest tissue that is thin and wide in humans, with a surface of about 2 m^2^ and 4 kg (1). The skin is mainly composed of the epidermis and dermis, separated by the junction of the two parts. Skin is essential to human health as it is the body's first line of defense against harmful external physical, chemical, and biological invasions, as well as other functions, such as controlling body temperature. In addition, skin is not only regarded as a physical barrier, but also a dynamic tissue with its metabolism and the interaction between internal and external cells (1). As external environment changes, coupled with a fast-paced lifestyle, environmental pollution, abuse of hormone products, contamination of pesticide residue, and increased mental stresses, a series of skin-related problems have increasingly occurred, including dryness, roughness, aging, and dull skin tone, leading to the chronic sub-health of the skin (2). Previous studies have shown that natural active substances from plants could effectively improve skin health. Chowdhury found that polyphenols like EGCG could increase extracellular matrix deposition of elastin and collagen and may improve skin properties (3). Kang reported that marigold methanol extract with high levels of polyphenols has significant skin anti-aging potential (4). Amakye et al. reported that two peptides obtained from soybean, Trp-Pro-Lys (WPK) and Ala-Tyr-Leu-His (AYLH), have better anti-aging effects compared with oyster peptides and sea cucumber peptides (5).

Selenium (Se), as an essential micronutrient for human health with many physiological activities, possesses a superior antioxidant and detoxifying effect on the skin. Se is one of the four internationally recognized antioxidants that promotes the breakdown of peroxides and protects hemoglobin in cells from oxidative damage (6). Burke et al. found that selenomethionine has been shown to protect subjects from acute skin damage following UVB exposure (7). Michaelssons et al. have demonstrated that a good clinical result was obtained after supplementation of Se in male acne patients (8). Organic Se exhibits more biological activity, relatively less toxicity and side effects than inorganic Se, whereas plants or soil microorganisms can transform inorganic Se into organic Se (9).

Mung bean (*Phaseolus radiatus* L.) is one of the important legumes widely planted in temperate and subtropical regions. Studies have shown that mung bean (MB) possesses a variety of potential bioactivities like hypolipidemic, antioxidant, anti-tumor, antibacterial, and immune enhancement (10), which could be attributed to the various nutrients (including protein, lipids, minerals, vitamins, and carotene) and functional bioactive ingredients (such as alkaloids, flavonoids, coumarins, and functional oligosaccharides) in MBs (10–12). Since both Se and MB possess vital effects on human health, Se-MBs can optimize the physiological and pharmacological functions of two components. However, most people currently consume MBs as food, and after passing through the human digestive system, many active ingredients (such as polyphenols) are metabolized and destroyed by several digestive enzymes, leading to the loss of their biological activity. Besides, the biological value of the nutrients in MB may be limited by some of the anti-nutritional factors it contains (13). For instance, phytic acid in MB can combine several important divalent cations, such as zinc, iron, magnesium, and calcium to form insoluble complexes to limit the absorption and utilization of minerals in the small intestine (14). Fortunately, these disadvantages can be reduced or eliminated through various processing methods, such as sprouting, fermentation, etc. (13).

Since the cell walls of Se-MB are difficult to break (the main components are cellulose and pectin), the various active substances in the cells cannot be fully utilized. Biological fermentation can be used to rupture the cell walls of tissues and break down macromolecular substances into small molecular substances, thereby releasing a variety of nutrients or active substances (15). However, one major drawback of current biological activity research of MB is that there is rare research on skin protection activity, which limits the research of MB. In this study, we used *Lactobacillus helveticus* and *bifidobacterial* to prepare Se-MBFB by fermenting selenium-enriched mung beans. The chemical composition was analyzed by measuring the content of total polyphenols, total flavonoids, peptides, and free amino acids to explore the whitening, moisturizing, and anti-aging activity of Se-MBFB. Besides, we use the Se-MBFB in the preparation of facial masks, which provides a reference for the development of Se-MB and its related industrial cosmetics products.

## Materials and Methods

### Materials and Reagents

Se-MBs (Se content: 2.83 ± 0.13 mg/kg) were derived from En Shi City (Hubei Province, China). The mouse melanoma cell line (B16F10) and human foreskin fibroblasts (HFF-1) were purchased from the National Collection of Authenticated Cell Cultures (Shanghai, China). Se standard solution was provided by the Shanghai Institute of Measurement and Testing Technology (Shanghai, China). The 0.25% Trypsin-EDTA, fetal bovine serum (FBS), DMEM, and RPMI 1640 culture mediums were obtained from Gibco Co. (California). The Se-MBFB facemask was commissioned to be produced by Shanghai Guzidi Industrial Co., Ltd (Shanghai, China), its formula is listed in Supplementary Table S1, the addition of other raw materials except Se-MBFB was to improve the experience of the subjects and did not have any efficacy. The reverse transcription and real-time PCR kits were purchased from Takara (Otsu, Japan). Tyrosinase, L-3,4-dihydroxyphenylalanine (L-DOPA) was obtained from Shanghai Baomanbio Technology Co., Ltd (Shanghai, China). 2,2-diphenyl-1-picrylhydrazyl (DPPH) was provided by Seebio Biotech Co., Ltd (Shanghai, China). 2,2-azino-bis-(3-ethyl-benzothiazoline-6-sulfonic acid) (ABTS) and vitamin C (Vc) were obtained from Sinopharm Chemical Reagent Co., Ltd (Shanghai, China). *Lactobacillus helveticus* and *Bifidobacteria* (*HN019*™) were obtained from Shanghai University (China). All other chemicals and solvents were of analytical grade.

### Preparation of Se-MBFB

The Se-MBFB was prepared as described earlier by Song with modification (16). Se-MBs were treated with 75% (w/w) or (w/v) aqueous ethanol solution for 1 to 2 s, washed three times with sterile distilled water, and then soaked for 24 h until sprouted to 5–6 mm. A total of 60–70% sterile distilled water, 10% Se-MBs (after pretreatment), and 2% of *Lactobacillus helveticus* and *Bifidobacteria* (*HN019*™) (1:1) liquid were added into the fermenter, and the nitrogen was used to remove oxygen in the fermenter. The fermentation was conducted at 37°C for 48–50 h, with stirring for 2 min per 8 h to make fermentation uniform. After the end of the fermentation, the fermentation broth was filtered with a microporous membrane and stored at 4°C, and the Se-MBFB was obtained.

### Determination of Main Active Ingredients in Se-MBFB

#### Total Amino Acid Composition Analysis

A total amino acid composition of free peptides and protein hydrolysate was determined as described earlier by Shazly et al. with slight modifications (17). A total of 1 mL of Se-MBFB was mixed with 2 mL of 6 mol/L HCl and 20 μL of phenol and then blown with nitrogen for 5 min. Thereafter, the mixture was hydrolyzed at 110 ± 1°C for 22 h, cooled to room temperature, and centrifuged (12,000 rpm, 4°C, 25 min). A total of 600 μL supernatant was dried with nitrogen and dissolved in 400 μL of HCl (0.02 mol/L). After filtration with 0.22 μm membrane, the supernatant was analyzed with an L-8900 automatic amino acid analyzer (Hitachi, Tokyo, Japan).

#### Free Amino Acids Composition Analysis

A total of 1 mL of Se-MBFB was mixed with 500 μL of 0.1 M HCl and 500 μL 10% trichloroacetic acid solution, and then reacted at 4°C for 0.5 h and centrifuged for 25 min (12,000 rpm, 4°C). A total of 200 μL supernatant was filtered with 0.22 μm membrane and determined with L-8900 automatic amino acid analyzer (Hitachi, Tokyo, Japan).

#### Determination of Chemical Composition

The content of polysaccharides was determined by the phenol sulfuric acid method, and glucose was used as a standard. The content of polyphenols was measured by Folin-Ciocalteu's reagent method according to the Chinese National Standard. The content of flavonoids was estimated according to the Chinese National Standard with rutin as a standard. The peptide content was determined by the Biuret method (18). The protein content was measured according to the Bradford method based on the standard curve of BSA (19). The peptide molecular weight was determined by the HPLC system (20). The total selenium content was determined by the Ventura method (21).

### Whitening Activity Assays *in vitro*

#### Inhibitory Effect on the Tyrosinase Activity

The composition of the reaction system with a total volume of 4 mL was as follows: 1 mL of L-DOPA as the enzyme-substrate (1.5 mg/mL, dissolved in pH 6.8 PBS), various concentrations (0.25, 0.5, 1, 2, 4, 8%) of Se-MGFBs, 0.5 mL of tyrosinase solution (200 U/mL, dissolved in pH 6.8 PBS) pre-stored at 37°C to maintain the optimum enzyme activity, and corresponding volume of PBS solution (pH 6.8). Then L-DOPA, Se-MGFBs, and tyrosinase were mixed and pre-incubated at 37°C for 10 min. After that, L-DOPA was added and incubated at 37°C for 5 min and then measured under 475 nm. The PBS was used instead of the sample solution as a negative control, and Vc was used as a positive control. The inhibition activity of the tyrosinase enzyme was calculated as follows:


(1) $$\begin{array}{l} \text{Tyrosinase inhibition }(\%)\text{ = }(\text{1}-{\text{OD475}}_{\text{sample}}{\text{/OD475}}_{\text{negative}})\\ \;\times 100\% \end{array}$$

#### Effect on Melanin Formation

The murine B16F10 melanoma cell line was maintained in RPMI1640 medium supplemented with 5% fetal bovine serum and Gentamicin (50 μg/mL) at 37°C in a humidified atmosphere containing 5% CO_2._

##### Cell Viability Assay

Cell viability was determined using the MTT assay. Briefly, the B16F10 cells were seeded at a density of 2 × 10^4^ cells/well in a 96-well tissue culture plate. After 24 h of incubation, the cells were treated with various concentrations (0.5, 1, 2, 4, 8, and 16%) of Se-MGFBs for 24 h. After media removal, fresh media containing MTT solution (1 mg/mL) was added and incubated for 4 h at 37°C. Subsequently, the formazan crystals formed were dissolved in 150 μL DMSO and vibrated at a low speed for 15 min, then the absorbance was measured at 570 nm using the AMR-100 microplate reader (Olympus, Tokyo, Japan), and the group without samples was regarded as the control.


(2) $$\begin{array}{r} \text{Cell viability }\left(\text{\%}\right)=\text{OD}570_{\text{sample}}/\text{OD}570_{\text{control}}\;\times \;100\text{\%} \end{array}$$

##### Inhibition Assay of Melanin Production

The melanin content in murine melanoma cells was measured using a previously described method with slight modifications (22). Briefly, B16F10 cells were cultured at a density of 5 × 10^4^ cells/well in a 6-well plate for 24 h, then the cells were treated with Se-MBFBs (2, 4, 8%). After 48 h of incubation, the cells were harvested and washed twice with PBS. The pelleted cells were dissolved in 200 μL of NaOH (1 mol/L), incubated at 100°C for 2 h, and then measured at an absorbance of 405 nm. The group without samples was regarded as control, Kojic acid (25 μg/mL) was a positive control, and the group without cells was regarded as blank. The relative content of melanin was calculated according to the following formula:


(3) $$\begin{array}{l} \text{Relative content of melanin }(\%)\text{=}({\text{OD405}}_{\text{sample}}{\text{/OD405}}_{\text{blank}})\\ \;\times 100\% \end{array}$$

### Moisturizing Activity Assays *in vitro*

HFF-1 cells were maintained in DMEM medium supplemented with 10% FBS and cultured in a carbon dioxide incubator with constant temperature (5% CO_2_, 37°C).

#### Cell Viability Assay

The cell viability was determined using different concentrations of sample (0.78, 1.56, 3.13, 6.25, 12.5, 25%) according to the description by Section Cell Viability Assay).

#### Cell Moisturizing Gene Expression Assay

The HFF-1 cells were cultured at a density of 2 × 10^4^ cells/well in a 12-well plate for 48 h, then the cells were treated with Se-MBFBs (1.56, 3.13, 6.25%). After 48 h of incubation, the cells were harvested and washed once with PBS, then total mRNA in HFF-1 cells was extracted by RNA Extraction kit according to the manufacturer's instructions, then total mRNA was reverse transcribed into cDNA according to the Reverse transcription kit instructions. The mRNA expression level was detected by SYBR qPCR Master Mix (Thermofisher, Beijing, China) according to the light quantitative PCR kit instructions. The specific primers used were as follows: AQP3, 5′-CCTTTGGCTTTGCTGTCACTC-3′(F), 5′-ACGGGGTTGTTGTAAGGGTCA-3′(R); GAPDH, 5′-AAGAAGGTGGTGAAGCAGG-3′(F), 5′-AGGTGGAGGAGTGGGTGTCG-3′(R). The gene GAPDH was employed as an internal reference and the relative mRNA level of target genes was calculated by using the 2^−Δ*ΔCt*^ method.

### Anti-aging Assays *in vitro*

#### DPPH Radical Scavenging Assay

Different concentrations of Se-MBFB (1, 2, 4, 8, and 16%) were mixed with 2 mL of 0.1 mM DPPH (dissolved in 95% ethanol solution) at room temperature for 30 min. The absorbance value was determined at 517 nm and recorded as A1. The sample solution was replaced by an equal volume of 95% ethanol and distilled water, and their absorbance values were recorded as A0 and A2, separately, and Vc was used as a positive control. The DPPH radical scavenging rate of the sample was calculated as follows:


(4) $$\begin{array}{l} \text{DPPH radical scavenging rate }(\%)\text{ = }[\text{A0}-(\text{A1}-\text{A2})]\text{/A0}\\ \;\times 100\% \end{array}$$

#### ABTS Radical Scavenging Assay

A total of 10 mL ABTS (7 mmol/L) solution and equal volume K_2_S_2_O_8_ (2.45 mmol/L) solution were mixed and kept in the dark for 12–16 h at room temperature, then diluted 40–50 times with 95% ethanol to make the absorbance reach 0.70 ± 0.02 at 734 nm and obtain ABTS test solution. A total of 4.0 mL ABTS test solution was added into Se-MBFBs (1, 2, 4, 8, and 16%) with different concentrations and incubated at room temperature for 10 min, the absorbance at 734 nm was measured and recorded as A1. The absorbance value of 95% ethanol instead of sample solution was recorded as A0, and Vc was used as a positive control. The ABTS radical scavenging rate of the sample was calculated as follows:


(5) $$\begin{array}{r} \text{ABTS radical scavenging rate }\left(\text{\%}\right)=\left(1-\text{A}1/\text{A}0\right)\times 100\text{\%} \end{array}$$

### Safety Evaluation

According to the Safety and Technical Standards for Cosmetics (STSC, version in 2015, China), heavy metal content analysis, skin sensitization test, acute eye/corrosion irritation test, and dermal irritation/corrosion test were performed following the criteria to evaluate its safety.

### Whitening, Moisturizing, and Antioxidant Activities *in vivo*

A total of 31 female volunteers have been enrolled upon inclusion/exclusion criteria screening by the investigator, they were aged between 25 and 60 years (tested by Intertek Shanghai Healthcare and Beauty Product Clinical Research Service Lab). Volunteers were excluded if they had pigmentation disorders, acute or chronic illness, and history of hypersensitivity reaction to any ingredient of the tested products. Also, volunteers who have used a whitening, moisturizing, or antioxidant product on the investigated areas within the month before the study and/or during the experiment were also excluded. Besides, volunteers under medical treatment for skin disease in the past and/or present with a therapy that may influence the results of the study were excluded. Before the start of the test, all subjects were given test instructions and signed informed consent.

The test environment was conducted under a condition with constant temperature and humidity (temperature: 21.0 ± 1.0°C; humidity: 50.0 ± 10.0%). The volunteer's face was cleaned with sterile pure water, then they sat quietly for 30 min. After that, various indicators of the face were analyzed and tested. The volunteers used Se-MBFB masque on their face every 2 d, 15–20 min each time, then cleaned their face with sterile pure water. The study lasted for 4 weeks, with visits at weeks 0, 2, and 4 (W0, W2, and W4). Clinical assessments and instrumental measurements were performed at every visit, and target sites were cheek and canthus, the following parameters were recorded on the target sites at each visit: Melanin index (Mexameter, Courage & Khazaka, Germany) and skin color (L^*^b^*^ system; chroma meter CR-400, Japan). Cuticle moisture content (Corneometer, Courage & Khazaka, Germany) and transepidermal water loss (Tewameter, Courage & Khazaka, Germany). Also, the center, right, and left sides of the face were photographed separately using the VISIA-CR (Canfield, OH) imaging station, and crow's feet were determined by Primos (LMI Technologies Gmbh, Canada). Besides, a study that included 26 healthy female volunteers aged 25–35 was performed to evaluate the short-acting moisturizing effect. The cuticle moisture content of the forearm was assessed by Corneometer (Courage & Khazaka, Germany) after using the mask 1 h, 6 h, and 8 h. All tests are conducted in accordance with the instructions of the instrument.

### Statistical Analysis

The data were expressed as the mean ± standard deviation (SD; *n* = 3) and analyzed using the GraphPad Prism 8.0 Software (GraphPad Software, Inc., San Diego, CA, USA). Analysis of variance (one-way, ANOVA) was performed using SPSS software (version 20.0). Since sample variance was homogeneous, Duncan's test (parametric) was used to identify statistically different samples. A value of *p* < 0.05 was considered significant.

## Results

### Chemical Composition Analysis of Main Active Ingredients in Se-MBFB

The total selenium content of Se-MBFB is 0.003 ± 0.001 (ppm). Table 1 presents the experimental data on some main active components content. As shown in Table 1, the content of polyphenols was the highest, followed by the peptides, proteins, and flavonoids, and the polysaccharides content was the lowest. Our results indicated that Se-MB contained functional active polyphenols which were strongly released and produced a high content of new functional active peptides (6.90 mg/mL). In addition, the molecular weight of most active peptides was small (Supplementary Figure S1).

**Table 1:** Chemical composition of Se-MBFB.

**Sample**	**Unit (mg/mL)**				
	**Polysaccharides**	**Polyphenols**	**Flavonoids**	**Peptides**	**Proteins**
Se-MBFB	0.26 ± 0.02	26.11 ± 0.56	0.52 ± 0.02	6.90 ± 0.61	3.24 ± 0.32

### The Amino Acids Composition Analysis

**Figure 1:**
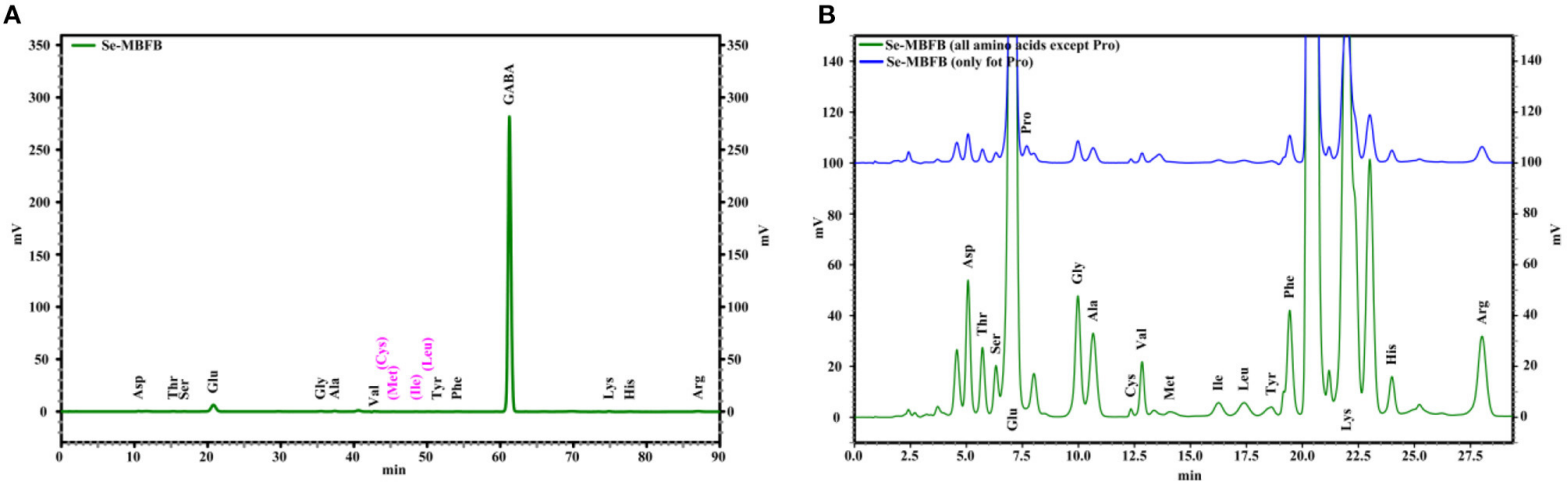
The IC chromatograms of the amino acids of free amino acid **(A)** and free peptides and protein hydrolysate **(B)** in Se-MBFB.

The amino acid compositions of free amino acids and peptides in Se-MBFB are presented and summarized in Figure 1 and Table 2. Briefly, 13 free amino acids with small molecular weight were obtained from Se-MBFB (Figure 1A), and γ-aminobutyric acid (GABA), Glu, Arg, Ala, and Gly had the top five amino acid contents. Among them, GABA was the dominant amino acid among the free amino acids in Se-MBFB, and its content was much higher than other amino acids (94.81%). Free peptides and protein hydrolysate were composed of 17 amino acids (Figure 1B), including 7 essential amino acids. Among them, Glu, Lys, Arg, Gly, and Ala were the top five amino acids.

**Table 2:** The amino acids of free amino acid and peptides in Se-MBFB (nmol%).

**Category**	**Free amino acid**	**Amino acid of free peptides and protein hydrolysate**
Asp	0.14	3.44
Thr	0.1	1.73
Ser	0.04	1.30
Glu	3.77	47.00
Gly	0.17	4.22
Ala	0.24	3.87
Cys	–	0.14
Val	0.17	1.45
Met	–	0.30
Ile	–	0.71
Leu	–	0.85
Tyr	0.03	0.55
Phe	0.07	3.54
Lys	0.13	23.07
His	0.05	1.38
Arg	0.29	4.38
Pro	–	2.07
GABA	94.81	–

‘*–'Indicated that the content was lower than the detection limit and not detected*.

### Evaluation of Whitening, Moisturizing, and Antioxidant Activities of Se-MBFB *in vitro*

#### Whitening Activity

**Figure 2:**
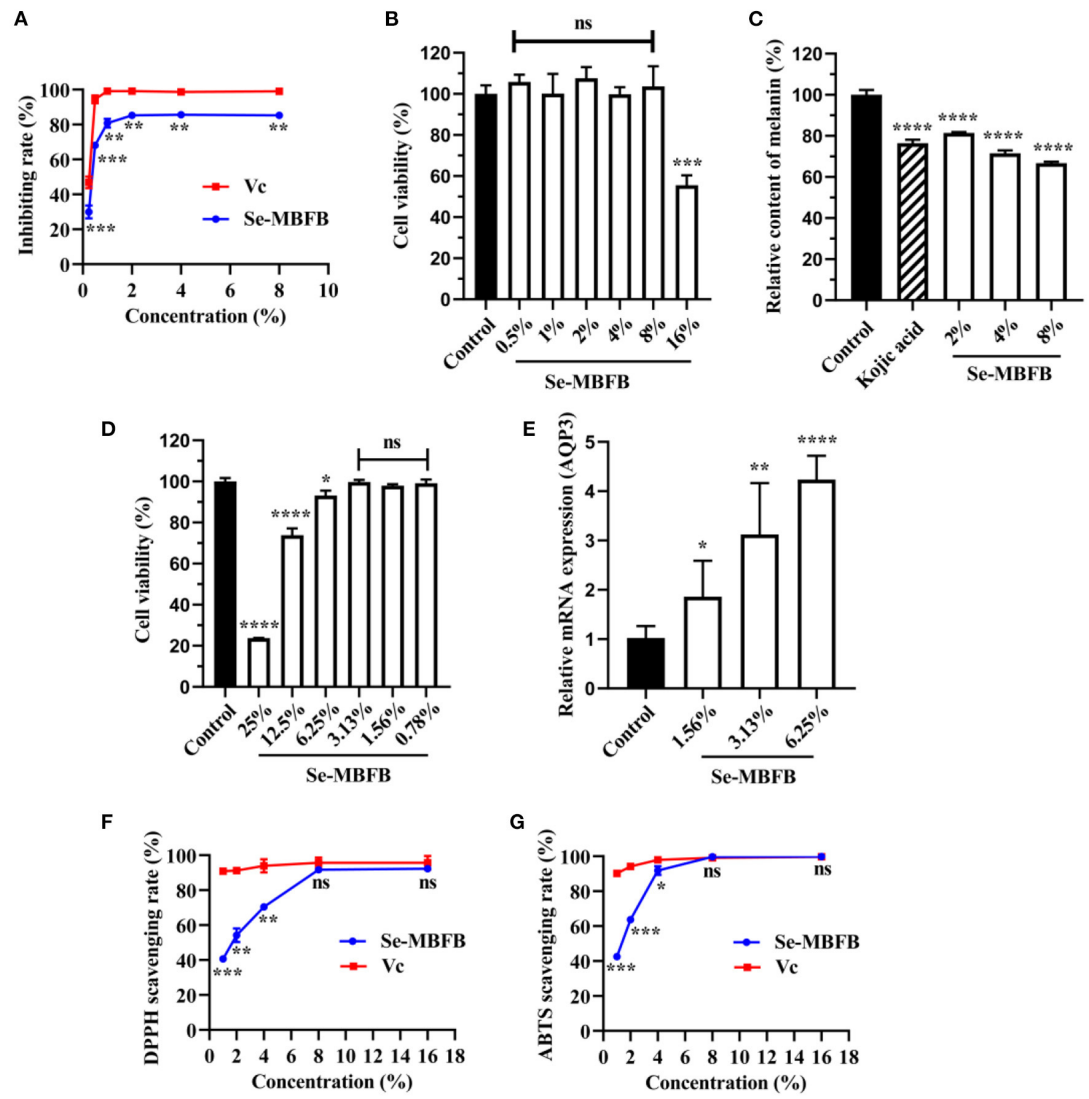
**(A)** Comparison of tyrosinase inhibition rates between Se-MBFB and Vc; **(B)** effects of Se-MBFB on B16-F10 cell viability; **(C)** effects of Se-MBFB on melanin synthesis in B16-F10 cells; **(D)** effects of Se-MBFB on HDF cell viability; **(E)** effect of Se-MBFB on expression of AQP3 in HDF cells; **(F)** effect of Se-MBFB on DPPH free radical scavenging activity; **(G)** effect of Se-MBFB on ABTS free radical scavenging activity. A significant difference was presented with *p* < 0.05 compared with control or Vc group, ‘*' represented *p* < 0.05, ‘**' represented *p* < 0.01, ‘***' represented *p* < 0.001, and ‘****' represented *p* < 0.0001.

Just as shown in Figure 2A, the Se-MBFB exhibited an outstanding tyrosinase inhibition rate, notably lower than Vc but more than 80% at high concentration. Se-MBFB could inhibit the activity of tyrosinase in a dose-dependent manner, which implies that Se-MBFB may be a promising new skin whitening ingredient.

The viabilities of cells treated with Se-MBFB (0.5–16%) were determined using the MTT assay. The outcome demonstrated that there was no significant cytotoxic effect under 16% of Se-MBFB (Figure 2B). Three doses of Se-MBFB (2, 4, and 8%) were used to investigate the effect of Se-MBFB on melanin production. The melanin synthesis assay displayed that Se-MBFB showed a consistent and significant reduction in the melanin content of B16F10 mouse melanoma cells in a dose-dependent manner (Figure 2C). The data showed that melanin content of cells treated with Se-MBFB was even lower than Kojic acid, further indicating that Se-MBFB has a potential whitening effect.

#### Moisturizing Activity

The viabilities of cells treated with Se-MBFB (0.78–25%) were determined using the MTT assay. The experimental results showed that the Se-MBFB affected the cell viability of HDF cells in a dose-dependent manner (Figure 2D). The concentrations of 6.25, 12.5, and 25% were significantly cytotoxic to cells, but considering the small effect of 6.25% of Se-MBFB on cell viability (>95%) and the potential moisturizing effect of higher concentrations, 6.25, 3.13, and 1.56% of Se-MBFB were used to investigate the moisturizing effect of Se-MBFB. The result exhibits that Se-MBFB prominently and dose-dependently promoted the expression of the moisturizing related gene, AQP3 (Figure 2E). It can be inferred that the Se-MBFB has potential moisturizing effects.

#### Anti-aging Activity

Since excessive free radicals can damage cells and accelerate the aging process of the human body (23), DPPH and ABTS scavenging assays were conducted to evaluate the anti-aging activity of the Se-MBFB. The results demonstrated that DPPH and ABTS radicals were eliminated dose-dependently by Se-MBFB (Figures 2F,G). Compared with Vc, the Se-MBFB possessed similar inhibition activity at high concentrations (no significant difference between them), which reveals that the Se-MBFB may have effective anti-aging activity.

### Safety Evaluation of Se-MBFB

No potential irritant was presented by heavy metal analysis (Supplementary Table S2), skin sensitization test (Supplementary Table S3), acute eye/corrosion irritation test (Supplementary Table S4), and dermal irritation/corrosion test (Supplementary Table S5), suggesting the potential of Se-MBFB for the development of safe facial skincare products.

### Whitening, Moisturizing, and Anti-aging Activities of Se-MBFB Facemask *in vivo*

#### Whitening Efficacy Evaluation

**Figure 3:**
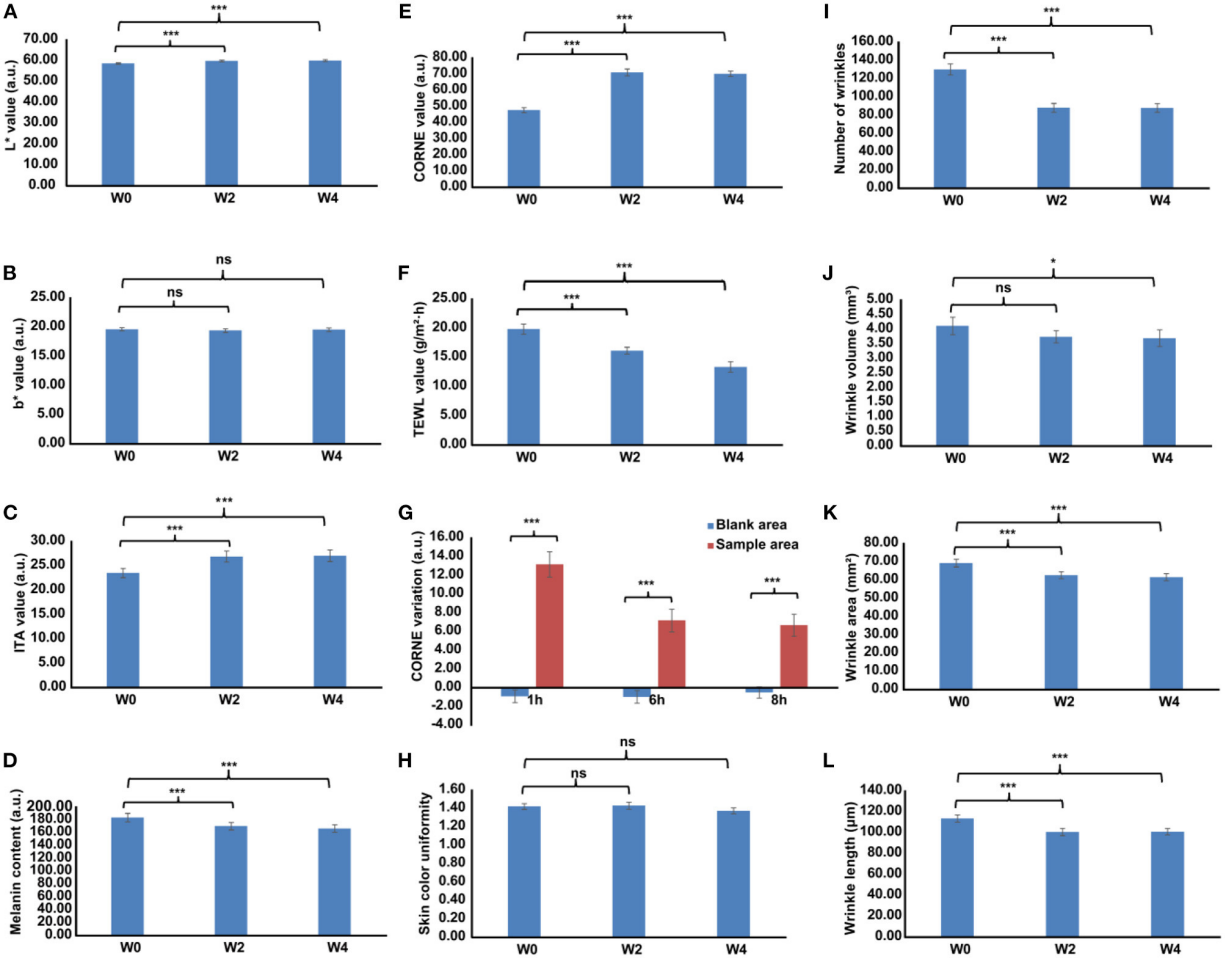
The whitening, moisturizing, and anti-aging activities of Se-MBFB facemask at 0, 2, and 4 weeks, differences adjusted by start values at W0. **(A)** L* value on the cheek areas; **(B)** b* value on the cheek areas; **(C)** ITA° value on the cheek areas; **(D)** melanin index on the cheek areas; **(E)** CORNE value on the cheek areas; **(F)** TEWL value on the cheek areas; **(G)** TEWL variation value on the forearm areas; **(H)** skin color uniformity value on face; **(I)** number of wrinkles on the canthus areas; **(J)** wrinkle volume on the canthus areas; **(K)** wrinkle area on the canthus areas; **(L)** wrinkle length on the canthus areas. L*, skin clarity; b*, skin yellowness; ITA°, individual typology angle; and MI, melanin index; CORNE, cuticle moisture content; TEWL, transepidermal water loss. A significant difference was presented with *p* < 0.05 compared with W0, ‘ns' represented *p* > 0.05, ‘*' represented 0.01 ≤ *p* < 0.05, ‘**' represented 0.001 ≤ *p* < 0.01, and ‘***' represented *p* < 0.001.

**Figure 4:**
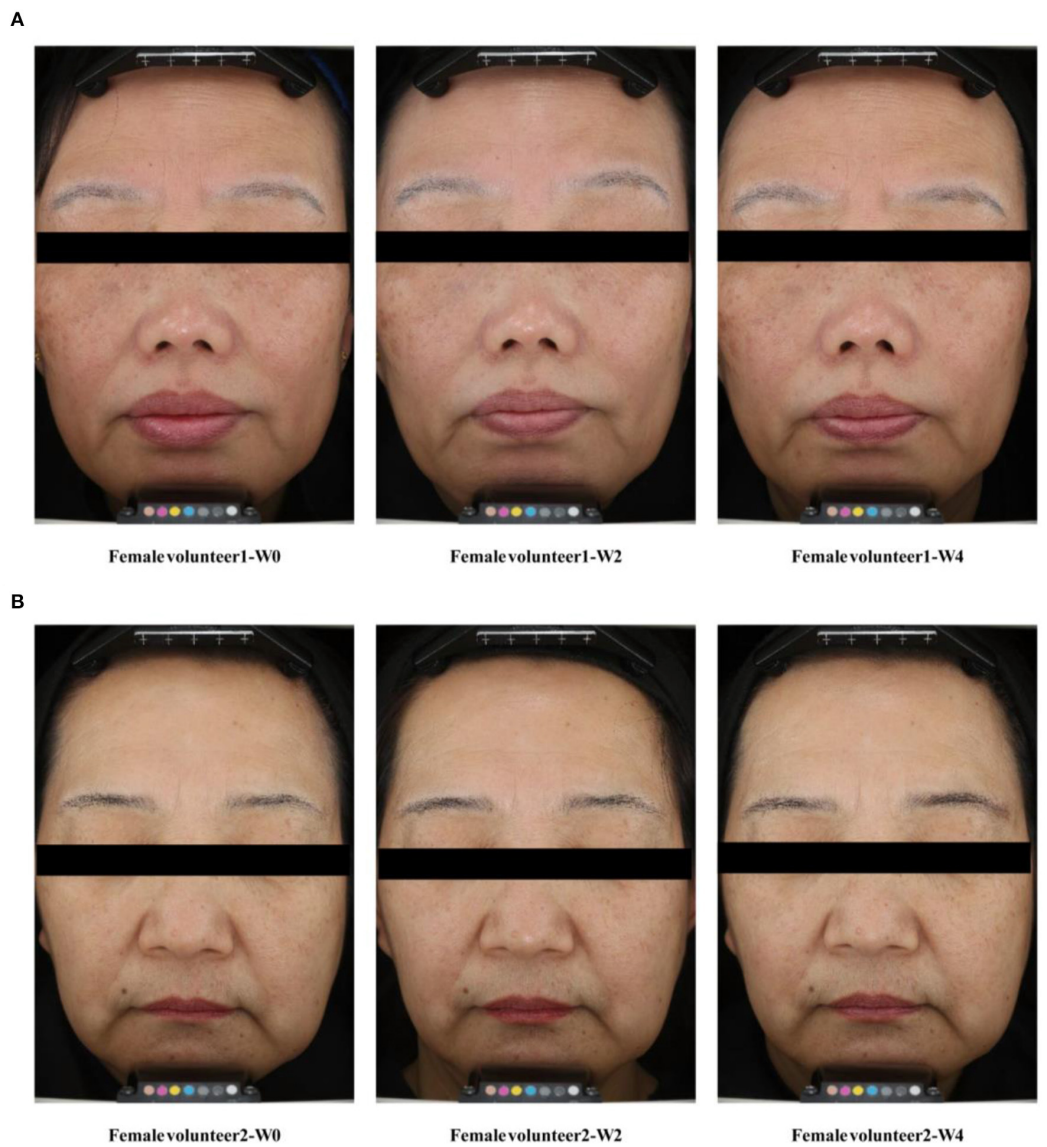
VISIA-CR facial image legend. **(A)** Case 1: Facial condition of volunteer 1 at 0, 2, and 4 weeks; **(B)** Case 2: Facial condition of volunteer 2 at 0, 2, and 4 weeks.

Over the study duration, L^*^ and ITA° values were markedly increased (Figures 3A,C), and the MI value was remarkably decreased after treatment with Se-MBFB facemask (Figure 3D). Although the b^*^ value was decreased, while there was no significant difference between W0 and W2 or W4 (Figure 3B). Compared with W0, there were significant changes in pigmentation level of hyperpigmented spots: L^*^ and ITA° increased at W2 and W4, and MI decreased greatly at W2 and W4. In addition, photographs taken by VISIA-CR at every visit demonstrated the changes in skin features, a gradual skin lightening color is depicted on the face (Figure 4). Thus, the Se-MBFB played a good role in whitening by making it less pigmented and melanogenesis.

#### Moisturizing Efficacy Evaluation

Over the 4-week study, cuticle moisture content was increased significantly and transepidermal water loss was decreased significantly (Figures 3E,F), while there was no significant change in skin color uniformity (Figure 3H). CORNE values at W2 and W4 were increased and TEWL values were significantly decreased compared with W0, indicating the Se-MBFB facemask can increase moisture in the skin and improve the skin barrier to prevent water loss in the skin. In addition, cuticle moisture content values increased significantly in the treated area (Figure 3G). From 1 h until the end of the study at 8 h, the hydration of the sample and control areas was decreased, respectively. However, cuticle moisture content values of the sample areas were more than the control areas, which suggests that the Se-MBFB facemask has a favorable effect on short-acting moisturizing.

#### Anti-aging Activity Evaluation

**Figure 5:**
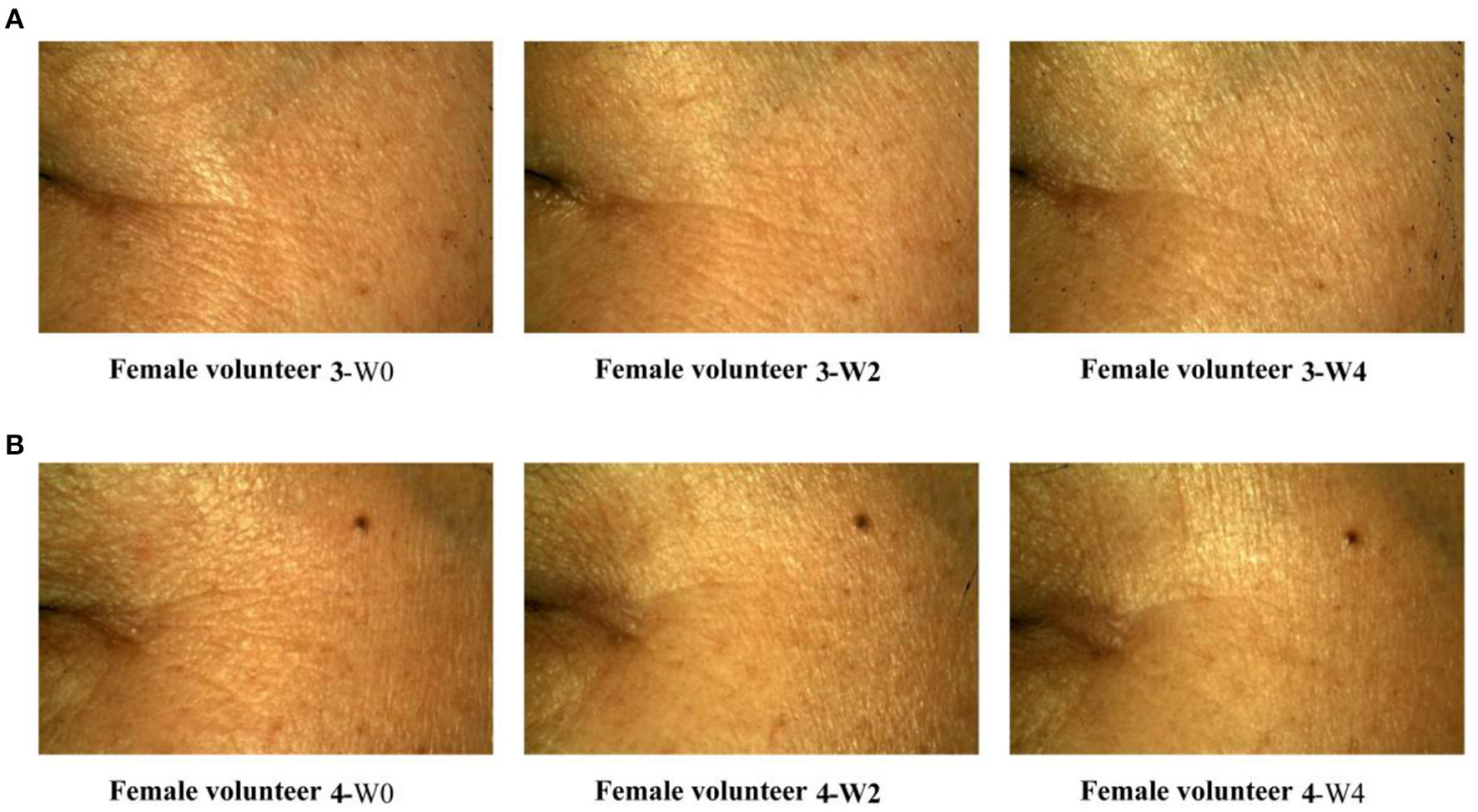
Primos crow's feet image legend. **(A)** Case 1: the crow's feet of volunteer 3 were observed at 0, 2, and 4 weeks; **(B)** Case 2: the crow's feet of volunteer 4 were observed at 0, 2, and 4 weeks.

On dermatome assessment of wrinkles (Figures 3I–L), the number of wrinkles, wrinkle volume, area, and length at W2 and W4 were significantly decreased compared with W0. Furthermore, the wrinkles of female volunteers were improved over time by using a facemask (Figure 5). These results prove that the Se-MBFB facemask possesses a good anti-aging effect.

## Discussion

Skin, as the body's first barrier, is an important part of maintaining human life and health. To the best of our knowledge, skin is extremely susceptible to damage caused by environmental factors. In addition to the aging factors brought about by age, stress response, pollution, sunlight, and ultraviolet radiation are all important causes of wrinkles in the skin (2). The aging of the social population and the deterioration of the environment will lead to increasingly serious aging of people's skin. Therefore, people will increasingly need long-lasting, efficient, regulatory, and preventive skincare products. Due to the few side effects and good curative effects of natural plants, skincare activities derived from natural plants have received extensive attention in recent years. In this study, we found that natural Se-MBFB had good whitening, moisturizing, and anti-aging effects on skincare with excellent safety. In addition, the facemask prepared by it also exerted good whitening, moisturizing, and anti-aging effects in human skin tests.

As a kind of legume food, MB contains rich nutrients, especially protein. It was reported that MB has been confirmed as an effective source of essential amino acids (deficient in many grains) (24). Thus, far, MB and its extracts have shown good biological activities. It was reported that the fermented MB had a greater effect on the regulation of blood glucose than the non-fermented MB (13). MB processed by boiling and sprouting showed higher hypolipidemic potential (13). Notably, the anti-melanogenesis become one of the hot spots of MB activity in recent years (13). In addition, the polysaccharides, polyphenols, and peptides contained in the mung bean exhibit antioxidative activity, which can improve the body's health. The health benefits of Se have been widely confirmed. It is worth noting that Se is a good antioxidant and anti-aging active substance (25). Therefore, Se-MB exhibited the synergistic health-promoting effects of Se and MB. The results showed that Se-MBFB showed significant whitening, moisturizing, and anti-aging properties after bio-fermentation with a dose-dependent effect. Similarly, Norlaily et al. found the content of free amino acids and soluble phenolic acid in the fermented MB has increased obviously, along with a significant increase in the content of GABA. Furthermore, the fermented MBs have better antioxidant immunomodulatory (26). However, Norlaily et al.'s research focused on the suppression of cancer by fermented mung beans and found that the active substances are mainly phenolic acids and free amino acids. Our studies have revealed the potential of fermented MB as a functional food to maintain skin health, which may be attributed to the polyphenols, polysaccharides, and peptides with a small molecular weight in the MB fermentation broth, as well as the rich amino acid composition (especially GABA).

Tyrosinase, as an oxidoreductase that is widely present in the human body, is the main rate-limiting enzyme in the process of melanin synthesis. Inhibition of tyrosinase activity will reduce the efficiency of catalyzing the synthesis of melanin, resulting in reduced melanin production (27). A previous study showed that the tyrosinase inhibitory ability of the ethanol extract of MB is the highest among the 16 kinds of legume ethanol extracts, and the extract also demonstrates a significant inhibitory effect on melanin production in B16F1 melanoma cells (28). In addition, the proanthocyanidins and condensed tannins in the seed coat of MB show a good inhibitory effect on the tyrosinase activity and melanogenesis of B16 mouse melanoma cells (29). In line with these studies, it was found that Se-MBFB showed a good inhibitory effect on tyrosinase activity and prevents melanin production in a dose-dependent manner, which was also confirmed by the Se-MBFB mask test. Combining these results, it was speculated that this might be related to the presence of some special phenolic compounds in MBs. It was reported that the two pure phenolic compounds isolated from the ethyl acetate extract of MB—vitexin and isovitexin—had excellent inhibitory effects on melanogenesis (30). Furthermore, based on molecular docking, previous studies have reported that proanthocyanidins or condensed tannins in the seed coat of MB could interact with tyrosinase driven by hydrogen bonds and hydrophobic forces, which might exert a crucial role in inhibiting the production of melanin (29).

AQP3, located in the basal layer, is an internal membrane protein responsible for transporting water and uncharged molecules on the cell membrane (31). It was reported that AQP3 could bring water, glycerol, and triglycerides from the sebaceous glands into the epidermis from the circulation, thereby preventing the epidermis from drying, which was very crucial for repairing the skin barrier and moisturizing of the epidermis (31). Free radicals are the normal product of the human body's oxidative metabolism. Nevertheless, excessive free radicals will attack normal cells, cause destructive damage to cells, and ultimately produce irreversible death, which leads to skin aging (32). To date, the moisturizing and anti-aging effects of phenolic substances have been proven. Myung et al. reported that phenolics can prevent wrinkle formation by reducing the levels of matrix metalloproteinase and inflammatory cytokines and increasing the expression of moisturizing factors and antioxidant genes (33). A previous study has reported that phenolic substances could significantly improve skin's fine lines and wrinkles, pigmentation, elasticity, firmness, and light damage (34). The moisturizing and anti-aging effects of small molecule peptides have also been reported (23). Studies have shown that the Glu content in peptides is positively correlated with the inhibition of lipid oxidation. Glu residue is a good metal chelator and free based scavenger attributing to its binding capacity of metal ions (35). Polar amino acids including Lys, Arg, and Gly residues in peptides may play a critical role in the free radical scavenging activity because of their carboxyl and amino groups in the side chains (36). Ala is a hydrophobic amino acid, and studies have shown that a higher content of hydrophobic amino acid can promote free radical scavenging and metal chelating activities of protein hydrolysate components (37). In this study, the results indicated that free peptides and protein hydrolysate in Se-MBFB might have good antioxidant activity. Although there are related studies on the AQP3 moisturizing gene, the relevant action mechanism is still unclear (31).

As for the anti-aging capacity of Se-MBFB, it was observed that higher phenols and peptides could scavenge free radicals, thereby reducing skin wrinkles and delaying aging, which confirmed that excessive free radicals could accelerate aging to a certain extent (23). In addition to phenols and peptides, the moisturizing and anti-aging effects of amino acids cannot be ignored. The unbalanced ratio of amino acids (components of protein) will lead to a decrease in skin protein synthesis. Furthermore, epidermal keratinocytes need amino acids to synthesize some antimicrobial peptides and kill pathogens (38). Amino acid substitution is indispensable in the skin because the shedding of stratum corneum cells will lose amino acids. GABA is a non-protein amino acid, which shows an obvious promoting-effect of skin healing, anti-wrinkle, and preventing skin aging, as well as inhibiting nerve activity, which can slow down the excitement of the sensory central nervous system, and prevent it from affecting the muscles, thereby improving expression lines and tightening the skin (39). In this study, GABA in Se-MBFB accounted for 94.81% of free amino acids, which provides effective anti-aging activity of Se-MBFB. Glu is the main constituent amino acid of peptides in Se-MBFB, which can be converted into GABA by Glutamate decarboxylase with anti-aging effects under certain conditions (40). Arg is a dibasic amino acid and the main component of keratin. It can accelerate the synthesis of human collagen tissue, promote wound healing, and has a significant moisturizing effect (41). Ala is a humectant added to some skincare products, which has a certain effect on the water retention of the stratum corneum. Gly is the constituent amino acid of the endogenous antioxidant reducing glutathione, which can condense with salicylaldehyde to inhibit the formation of hydroxyl free radicals in the body to achieve an antioxidant effect and better prevent skin aging (42). Due to their small molecular weight, these amino acids are easier to penetrate the epidermis of the skin and enter the dermis, thereby improving skin health (1).

The application of MB to the production of facial masks has been studied. Similarly, Husni et al. used MB in the production of facial masks because of its antioxidant activity (43). Husni et al. focused on optimizing the formulation of the mask and its physical properties while our study focuses on the activity of the mask. In this study, a mask with Se-MBFB as the main functional substance was designed. An Se-MBFB mask exerted a good anti-wrinkle effect, whitening effect, and moisturizing effect in volunteer trials, laying the foundation for the promotion and application of Se-MBFB.

## Conclusion

In this study, we prepared Se-MBFB through fermentation and conducted *in vitro* tests to reveal that Se-MBFB possessed good whitening, moisturizing, and antioxidant activities, and there were no safety hazards. The whitening effect was mainly reflected in the good inhibitory effect of Se-MBFB on tyrosinase activity. Phenols, peptides, and free amino acids were the main active substances of Se-MBFB for anti-aging and moisturizing. Moreover, an Se-MBFB facemask was prepared and a series of skin test experiments on it were designed, which further confirmed the skincare activity of Se-MBFB. Our results provide a good reference for the functional development and economic benefit improvement of Se-MB related agricultural products.

## Data Availability Statement

The raw data supporting the conclusions of this article will be made available by the authors, without undue reservation.

## Ethics Statement

The studies involving human participants were reviewed and approved by Intertek Shanghai Healthcare and Beauty Product Clinical Research Service Lab. The patients/participants provided their written informed consent to participate in this study.

## Author Contributions

KW: conceptualization and writing—original draft preparation. CG: figure preparation and data acquisition. JZ: writing—original draft preparation and figure preparation. YaW: investigation, methodology, and revision. MW: data acquisition. XH and MZ: methodology. XuW and JL: software. YuW: revision. XiW: supervision, reviewing, and editing. All authors contributed to the article and approved the submitted version.

## Funding

This study was funded by projects commissioned by enterprises and institutions (2019310031001329) of Shanghai Yuemu Cosmetics Co., Ltd., Shanghai, China. The authors are grateful for financially sponsored by Program of Shanghai Academic/Technology Research Leader (20XD1433500), Shanghai Agricultural Leading Talent Program, the National Natural Science Foundation of China (No. 32172223), and the National Key R&D Program of China (No. 2018YFC1604401).

## Conflict of Interest

MZ and JL were employed by Shanghai Yuemu Cosmetics Co., Ltd. XuW was employed by Enshi Selenium Impression Agricultural Technology Co., Ltd. Shadi Township. The authors declare that this study received funding from Shanghai Yuemu Cosmetics Co., Ltd., Shanghai, China. They instructed and assisted in the production method of Se-MBFM in this study. The remaining authors declare that the research was conducted in the absence of any commercial or financial relationships that could be construed as a potential conflict of interest.

## Publisher's Note

All claims expressed in this article are solely those of the authors and do not necessarily represent those of their affiliated organizations, or those of the publisher, the editors and the reviewers. Any product that may be evaluated in this article, or claim that may be made by its manufacturer, is not guaranteed or endorsed by the publisher.
