# Supplementary material for: The Whitening, Moisturizing, Anti-aging Activities, and Skincare Evaluation of Selenium-Enriched Mung Bean Fermentation Broth
Source: Front Nutr. 2022 Mar 18;9:837168. doi: 10.3389/fnut.2022.837168 (PMC8973414; doi:10.3389/fnut.2022.837168)
Supplement: Supplementary file 1 [file Table_1.DOCX]

***Supporting Information***

***The whitening, moisturizing and anti-aging activities of selenium-enriched*** ***mung bean fermentation broth (Se-MBFB) and the comprehensive skincare evaluation of its masque prepared***

*Kang Wei ^a^, Congyin Guo ^b^, Yang Wei ^a^, Jiangxiong Zhu ^a^, Meirong Wu ^b^, Xiaodong Huang ^c^, Jide Li ^c^, Xueyun Wang ^d^, Yuanfeng Wang ^b*^, Xinlin Wei ^a*^*

*^a^ School of Agriculture and Biology, Shanghai Jiao Tong University, 800 Dongchuan Road, Shanghai 200240, PR China*

*^b^ College of Life Sciences, Shanghai Normal University, 100 Guilin Road, Shanghai 200234, PR China*

*^c^ Shanghai Yuemu cosmetics Co., Ltd., 977 Shangfeng Road, Shanghai 200240, PR China*

*^d^ Enshi Selenium Impression Agricultural Technology Co., Ltd. Shadi Township, Hubei 445000, PR China*

*Corresponding author.

Tel.: + 86-21-34208533

Address: No. 800 Dongchuan Road, Minhang District, Shanghai 201100, China

E-mail: foodlab2010@163.com

**Table S1** The Se-MBFB facemask formula

| Number | Raw material | INCI | Content (%) |
| --- | --- | --- | --- |
| 6 | Water | Water | 86.55 |
| 2 | Butanedio | Butanedio | 4.00 |
| 3 | Se-MBFB | *Lactobacillus*/Bean seed extract/Sodium glutamate ferment filtrate | 2.00 |
| 4 | Dendrobium polysaccharide | *Dendrobium Candidum* stem extract | 2.00 |
| 5 | Avenanthramides | *Avena Strigosa* seed extract | 2.00 |
| 6 | Glycerol | Glycerol | 2.00 |
| 7 | p-Hydroxyacetophenone | p-Hydroxyacetophenone | 0.50 |
| 8 | Stephania tetrandra extract | *Stephania Tetrandra* extract | 0.40 |
| 9 | PE9010 | Phenoxyethanol/Ethylhexylglycerol | 0.20 |
| 10 | Hydrolyzed sclerotium gum | Hydrolyzed sclerotium gum | 0.15 |
| 11 | Carbopol U20 | Acrylates C10-30 alkyl acrylate cross polymer | 0.10 |
| 12 | Arginine | Arginine | 0.10 |

**Table S2** Heavy metal content in Se-MBFB

| Heavy metals | Se-MBFB (mg/kg) | STSC requirements (mg/kg) |
| --- | --- | --- |
| Hg | ND | <1 |
| As | 0.115 | <2 |
| Pb | ND | <10 |
| Cd | ND | <5 |

ND: not detected.

**Table S3** Dermal irritation test score

| Application days | Number of cavy | Stimulus response score | | | | | | | | |
| --- | --- | --- | --- | --- | --- | --- | --- | --- | --- | --- |
|  |  | Se-MBFB | | | |  | Control | | | |
|  |  | Erythema | Edema | | Score |  | Erythema | Edema | Score | |
| 1 | 4 | 0/4 | 0 | | 0 |  | 0 | 0 | 0 | |
| 2 | 4 | 0/4 | 0 | | 0 |  | 0 | 0 | 0 | |
| 3 | 4 | 0/4 | 0 | | 0 |  | 0 | 0 | 0 | |
| 4 | 4 | 0/4 | 0 | | 0 |  | 0 | 0 | 0 | |
| 5 | 4 | 0/4 | 0 | | 0 |  | 0 | 0 | 0 | |
| 6 | 4 | 0/4 | 0 | | 0 |  | 0 | 0 | 0 | |
| 7 | 4 | 0/4 | 0 | | 0 |  | 0 | 0 | 0 | |
| Average score of each animal every day | | | | | 0 | | | | | |

**Table S4** Acute eye irritation test score

| Rabbit ID | Observation parts | Eye irritation test score | | | | | | | |
| --- | --- | --- | --- | --- | --- | --- | --- | --- | --- |
|  |  | 1 h | | 24 h | | 48 h | | 72 h | |
|  |  | Se-MBFB | Control | Se-MBFB | Control | Se-MBFB | Control | Se-MBFB | Control |
| 1 | Conjunctiva | 0 | 0 | 0 | 0 | 0 | 0 | 0 | 0 |
|  | Iris | 0 | 0 | 0 | 0 | 0 | 0 | 0 | 0 |
|  | Corneal | 0 | 0 | 0 | 0 | 0 | 0 | 0 | 0 |
| 2 | Conjunctiva | 0 | 0 | 0 | 0 | 0 | 0 | 0 | 0 |
|  | Iris | 0 | 0 | 0 | 0 | 0 | 0 | 0 | 0 |
|  | Corneal | 0 | 0 | 0 | 0 | 0 | 0 | 0 | 0 |
| 3 | Conjunctiva | 0 | 0 | 0 | 0 | 0 | 0 | 0 | 0 |
|  | Iris | 0 | 0 | 0 | 0 | 0 | 0 | 0 | 0 |
|  | Corneal | 0 | 0 | 0 | 0 | 0 | 0 | 0 | 0 |

**Table S5** Skin allergy test score

| Group | Observation time（h） | Erythema level | | | | | Edema level | | | | | Number of ≥ 2 (%) |
| --- | --- | --- | --- | --- | --- | --- | --- | --- | --- | --- | --- | --- |
|  |  | 0 | 1 | 2 | 3 | 4 | 0 | 1 | 2 | 3 | 4 |  |
| Negative control (n=10) | 24 | 10/10 |  |  |  |  | 10/10 |  |  |  |  | 0 |
|  | 48 | 10/10 |  |  |  |  | 10/10 |  |  |  |  |  |
|  | 72 | 10/10 |  |  |  |  | 10/10 |  |  |  |  |  |
| Se-MBFB (n=20) | 24 | 20/20 |  |  |  |  | 20/20 |  |  |  |  | 0 |
|  | 48 | 20/20 |  |  |  |  | 20/20 |  |  |  |  |  |
|  | 72 | 20/20 |  |  |  |  | 20/20 |  |  |  |  |  |
| Positive control (n=10) | 24 |  |  | 10/10 |  |  | 10/10 |  |  |  |  | 100% |
|  | 48 |  | 1/10 | 9/10 |  |  | 10/10 |  |  |  |  | 90% |
|  | 72 |  | 2/10 | 8/10 |  |  | 10/10 |  |  |  |  | 80% |


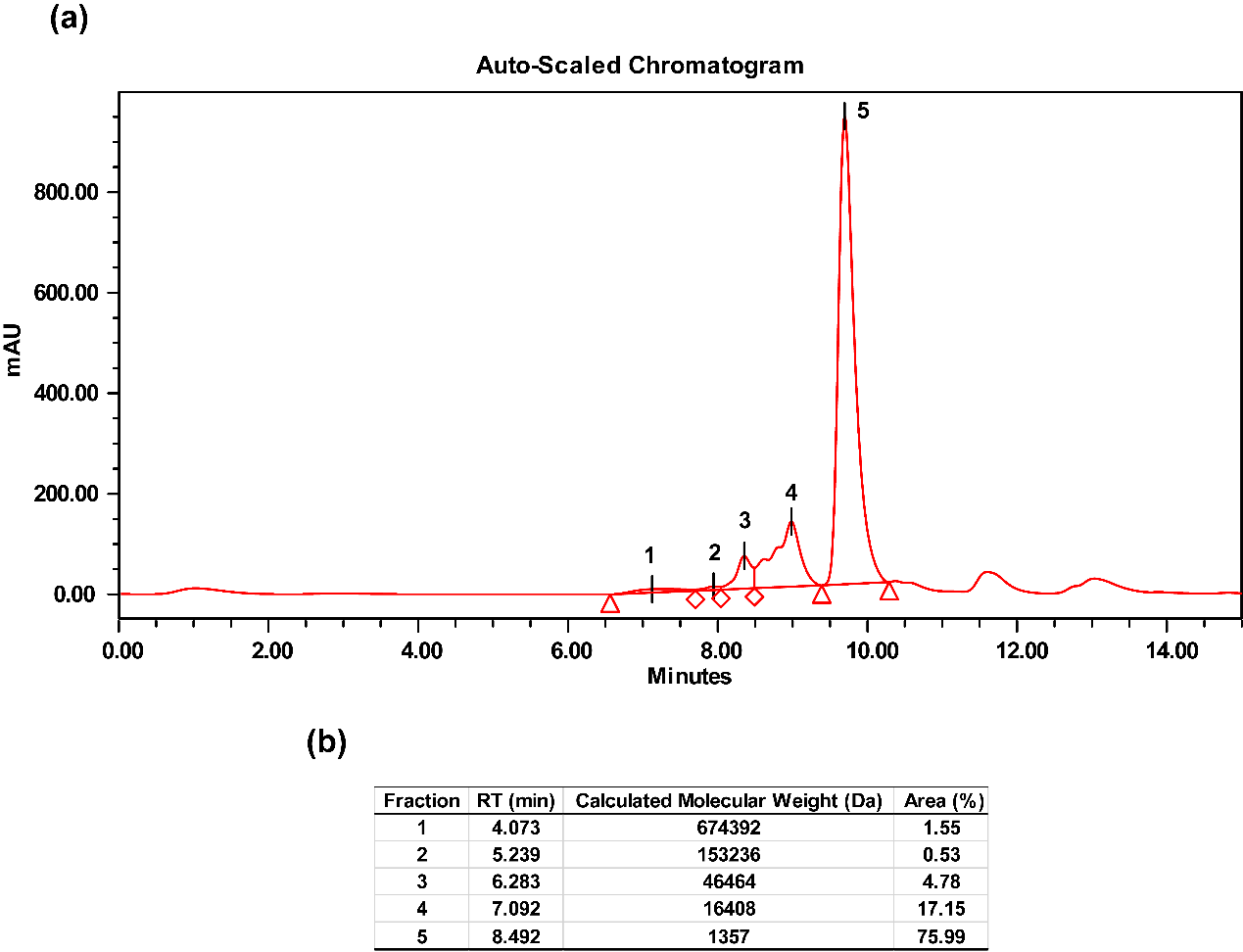


**Figure S1** (a) Chromatogram of Se-MBFB; (b) Molecular weight and content distribution of polypeptide in Se-MBFB.
